# Metastasis of hormone-independent breast cancer to lung and bone is decreased by α-difluoromethylornithine treatment

**DOI:** 10.1186/bcr1292

**Published:** 2005-08-09

**Authors:** Monica M Richert, Pushkar A Phadke, Gail Matters, Douglas J DiGirolamo, Sharlene Washington, Laurence M Demers, Judith S Bond, Andrea Manni, Danny R Welch

**Affiliations:** 1 Department of Pathology, Comprehensive Cancer Center, Center for Metabolic Bone Disease, National Foundation for Cancer Research – Center for Metastasis Research, University of Alabama at Birmingham, Alabama, USA; 2 Department of Biochemistry and Molecular Biology, The Pennsylvania State University College of Medicine, Hershey, Pennsylvania, USA; 3 Department of Medicine, The Pennsylvania State University College of Medicine, Hershey, Pennsylvania, USA; 4 Department of Pathology, The Pennsylvania State University College of Medicine, Hershey, Pennsylvania, USA

## Abstract

**Introduction:**

Polyamines affect proliferation, differentiation, migration and apoptosis of cells, indicating their potential as a target for cancer chemotherapy. Ornithine decarboxylase converts ornithine to putrescine and is the rate-limiting step in polyamine synthesis.

α-Difluoromethylornithine (DFMO) irreversibly inhibits ornithine decarboxylase and MDA-MB-435 human breast cancer metastasis to the lung without blocking orthotopic tumor growth. This study tested the effects of DFMO on orthotopic tumor growth and lung colonization of another breast cancer cell line (MDA-MB-231) and the effects on bone metastasis of MDA-MB-435 cells.

**Methods:**

MDA-MB-231 cells were injected into the mammary fat pad of athymic mice. DFMO treatment (2% per orally) began at the day of tumor cell injection or 21 days post injection. Tumor growth was measured weekly. MDA-MB-231 cells were injected into the tail vein of athymic mice. DFMO treatment began 7 days prior to injection, or 7 or 14 days post injection. The number and incidence of lung metastases were determined. Green fluorescent protein-tagged MDA-MB-435 cells were injected into the left cardiac ventricle in order to assess the incidence and extent of metastasis to the femur. DFMO treatment began 7 days prior to injection.

**Results:**

DFMO treatment delayed MDA-MB-231 orthotopic tumor growth to a greater extent than growth of MDA-MB-435 tumors. The most substantial effect on lung colonization by MDA-MB-231 cells occurred when DFMO treatment began 7 days before intravenous injection of tumor cells (incidence decreased 28% and number of metastases per lung decreased 35–40%). When DFMO treatment began 7 days post injection, the incidence and number of metastases decreased less than 10%. Surprisingly, treatment initiated 14 days after tumor cell inoculation resulted in a nearly 50% reduction in the number of lung metastases without diminishing the incidence. After intracardiac injection, DFMO treatment decreased the incidence of bone metastases (55% vs 87%) and the area occupied by the tumor (1.66 mm^2 ^vs 4.51 mm^2^, *P *< 0.05).

**Conclusion:**

Taken together, these data demonstrate that DFMO exerts an anti-metastatic effect in more than one hormone-independent breast cancer, for which no standard form of biologically-based treatment exists. Importantly, the data show that DFMO is effective against metastasis to multiple sites and that treatment is generally more effective when administered early.

## Introduction

Metastasis is the leading cause of death of breast cancer patients. As tumors progress from hormone-dependent to hormone-independent, their risk of metastasis increases and the treatment options decrease. There are currently no efficacious biologically-based therapies for the more aggressive hormone-independent breast cancers. It is therefore important to explore potential therapies that may be effective against both metastasis and growth of hormone-independent breast tumors.

Polyamines are organic compounds derived from decarboxylation of the amino acid ornithine. These compounds play a role in a wide variety of cellular functions including proliferation, differentiation, migration and apoptosis [1]. They are required for cell viability, as demonstrated by the lethality of ornithine decarboxylase (ODC) null mutations in mice [2]. ODC is the rate-limiting enzyme in polyamine synthesis: It converts ornithine to putrescine to begin the polyamine cascade. Overexpression of ODC in the immortalized, but non-tumorigenic, mammary epithelial cell line MCF10A results in a partially transformed phenotype as well as in the activation of ERK, which is often activated in breast cancers [3,4]. Previous studies demonstrated that increased ODC activity correlates with transition to hormone independence, increased anaplasia and metastasis. Increased ODC activity is an independent adverse prognostic factor for overall breast cancer survival in women with localized disease [5-7], suggesting that increased activity of the polyamine pathway may result in more aggressive, hormone-independent breast cancers.

α-Difluromethylornithine (DFMO) is an irreversible inhibitor of ODC. Treatment with DFMO results in decreased polyamine pools causing a cytostatic effect. Our laboratory previously demonstrated that DFMO treatment decreased lung metastasis from the estrogen receptor-negative/progesterone receptor-negative breast carcinoma cell line MDA-MB-435 xenografts, while having only a modest or absent effect on the growth of the orthotopic tumors [8,9]. Previous studies have shown that DFMO (0.5–3% in drinking water) was well tolerated in both rats and mice. The minimal toxicities of DFMO make it a suitable candidate for long-term treatments.

The studies described here were designed to address two questions. First, does DFMO reduce metastasis to sites other than lung? To address this question, we took advantage of a recently developed enhanced green fluorescent protein (GFP)-expressing variant of MDA-MB-435 [10,11]. Second, does DFMO affect orthotopic tumor growth and metastasis of another hormone-independent breast carcinoma? To test this question, we utilized the estrogen receptor-negative/progesterone receptor-negative MDA-MB-231 cell line. We present here that DFMO can decrease metastasis of MDA-MB-435 cells to the bone and can affect lung metastasis and orthotopic tumor growth of MDA-MB-231 cells.

## Materials and methods

### Cell lines

MDA-MB-231 and MDA-MB-435 human breast cancer cell lines were kindly provided by Dr Janet E Price at the University of Texas MD Anderson Cancer Center. They were cultured in DMEM/Ham's F12 medium supplemented with 5% fetal bovine serum, 1% non-essential amino acids, 1 mM sodium pyruvate, and were maintained at 37°C with 5% CO_2 _in a humidified atmosphere. The cells were passaged using 0.125% trypsin, 2 mM ethylenediamine tetraacetic acid in Ca^2+^/Mg^2+^-free Dulbecco's PBS.

### Metastasis assays

All animals were maintained under the guidelines of the IACUC of the University of Alabama at Birmingham under registered protocols. Female athymic mice (Harlan Sprague-Dawley, Inc., Indianapolis, IN, USA) were used for all studies.

To study bone metastasis, 4-week-old to 6-week-old female mice were injected intracardially with 3 × 10^5 ^GFP-labeled MDA-MB-435 cells in 0.2 ml ice-cold Hanks Balanced Salt Solution (HBSS). Seven days prior to injection, two groups of 10 mice each were randomly selected. One group remained untreated, while the second group was provided DFMO as a 2% solution in the drinking water *ad libitum *until 6 weeks post injection. This dose had been previously demonstrated as efficacious. The mice were euthanized and the femurs dissected away from the soft tissue. Bone metastases were visualized by fluorescence microscopy [10,11]. Brightfield and fluorescent photographs were analyzed using Sigma Scan (SPSS Inc., Chicago, IL, USA) to determine the total area of bone, the area of bone occupied by fluorescing tumor cells and the percentage of bone occupied by fluorescing tumor cells.

MDA-MB-231 breast cancer cells (2 × 10^5 ^cells in 0.2 ml HBSS) were injected intravenously into the lateral tail vein of 3-week-old to 4-week-old athymic mice to evaluate lung colonization as metastases from an orthotopically growing tumor are relatively rare from this cell line. Each treatment group consisted of 20 mice. DFMO treatment (2% in drinking water) was administered using three schedules beginning: 7 days prior to tumor cell injection, 7 days after tumor cell injection, or 14 days after tumor cell injection. DFMO was administered until the experiment was terminated (4–6 weeks post injection). Control animals did not receive DFMO. At termination, the lungs were removed and fixed in Bouin's fixative diluted 1:5 with neutral-buffered formalin. The number of lungs with surface metastases were determined, as well as the number of surface metastases per lung by examination under a dissecting microscope, as described elsewhere [12].

Orthotopic tumor growth was measured by injecting MDA-MB-231 breast cancer cells (5 × 10^5 ^cells in 0.1 ml HBSS) into the second thoracic mammary fat pad of 5-week-old to 6-week-old female athymic nude mice, as described previously [12]. DFMO treatment (2% in drinking water) began at the time of mammary fat pad injection or at 21 days post injection. The control group consisted of 17 mice, the group started on DFMO at the time of injection consisted of 20 mice, while three mice began treatment 21 days post injection. Tumor growth was monitored weekly by measuring the tumor length and width with a caliper and was reported as the mean tumor diameter as previously described [12]. Since DFMO retarded tumor growth, DFMO-treated mice were euthanized when average local tumor diameters reached approximately 1.5 cm. In a subsequent experiment, all animals were euthanized 6 weeks post tumor cell injection. To examine growth parameters, mice were injected with 100 mg/kg BrdU approximately 2 hours prior to euthanasia. Tumors were removed and divided into two portions. One aliquot was fixed in 10% neutral-buffered formalin and processed for histological analysis. The other aliquot was frozen in liquid nitrogen and stored at -70°C for analysis of polyamine levels as described [9,13].

### Polyamine levels

Athymic mice were injected subcutaneously (or in the mammary fat pad as already described) with 5 × 10^5 ^MDA-MB-231 cells in 0.1 ml HBSS. The comparison of orthotopic sites with ectopic sites was carried out in order to evaluate potential pharmacologic differences. The mice were separated into two animals per group. The control group was left untreated, while DFMO treatment began 7 days prior to tumor cell injection, on the day of injection, or 21 days post tumor cell injection. At 6 weeks post injection, tumors were removed and homogenized in 25 mM Tris-HCl buffer, pH 7.4, containing 0.1 mM ethylenediamine tetraacetic acid and 2.5 mM dithiothreitol. The homogenates were centrifuged for 30 min at 20,000 × *g*. An aliquot of the supernatant was used to measure ODC activity. The remaining aliquot was extracted with 0.6 N perchloric acid for 1 hour at 4°C prior to centrifugation at 15,000 × *g *for 15 min. The supernatant was used for polyamine determination. Polyamine levels were determined using high-pressure liquid chromatography as previously described [9,13].

### Quantification of bromodeoxyuridine incorporation

Incorporation of BrdU was determined using immunohistochemical staining. Briefly, the sections were deparaffinized using xylenes and ethanol, and were then treated with 3% hydrogen peroxide for 10 min, 0.1% trypsin for 30 min and 2 N HCl for 30 min with PBS washes between each treatment. The sections were blocked with 5% normal goat serum in 1% BSA, 0.05% Tween 20 and 0.1% NaN^3 ^in PBS followed by a 2-hour incubation at 37°C in a 1:1000 dilution of a monoclonal anti-BrdU antibody (B2531; Sigma, St. Louis, MO, USA). The sections were rinsed and then incubated for 1 hour at 37°C in a 1:5000 dilution of anti-mouse AlexaFluor Green. After the final washes, the sections were cover-slipped in Vectashield mounting media containing DAPI diluted 1:7 with non-DAPI-containing Vectashield mounting media. Five photographs (100 × magnification) from two sections of each tumor were counted for BrdU-labeled cells and the total number of labeled cells was graphed.

### Quantification of TUNEL staining

Apoptotic cells were identified using the Apoptag plus staining kit from Chemicon (S7101; Temecula, CA, USA) according to the manufacturer's instructions. Five photographs (63 × magnification) from two sections of each tumor were counted for TUNEL-labeled cells, and the total number of labeled cells was graphed.

### Semi-quantitative RT-PCR

Total RNA was isolated using Trizol (Invitrogen, Carlsbad, CA, USA) and 500 ng was used for RT-PCR with a SuperscriptIII/Platinum Taq One Step Kit (Invitrogen) as directed. Human glyceraldehyde-3-phosphate dehydrogenase primers (5'-GTGAAGGTCGGAGTCAACGGATT-3' and 5'-AGTGATGGCATGGACTGTGGTC-3') and hypoxanthine phosphoribosyl transferase primers (5'-CCAAAGATGGTCAAGGTCGC-3' and 5'-CTGCTGACAAAGATTCACTGG-3') were used to assess equal template loadings, and the linear range for each primer pair was determined independently. Human meprin α levels were determined with the following primers: 5'-ATCGGAGGCACGGCTGGCGTG-3' and 5'-GCCTGCCCTCATGGAGCTTACAG-3'. RT-PCR products were separated on a 1% TAE/agarose gel and quantified using a Stratagene Eagle Eye system with multiple integrations.

### Statistics

Comparisons between treatment groups and control-treated mice were made using SigmaStat statistical software (SPSS Inc.). For multiple group comparisons, one-way analysis of variance was performed, followed by the Student-Neumann-Keuls post-test. Results were considered statistically different if *P *< 0.05.

## Results and discussion

In normal tissues, ODC activity is increased by a variety of environmental and genetic factors associated with carcinogenesis, including ultraviolet light, asbestos and androgens. Increased ODC activity persists in and is associated with a wide variety of epithelial neoplasms including breast, skin, colon and prostate (reviewed in [1]). Together, these correlations suggest a role for ODC in tumor development and progression. Functional evidence supports this conclusion. In skin carcinogenesis models, ODC heterozygous knockout mice have significantly reduced tumor development [14].

Hormone-independent breast cancers are generally more aggressive and metastatic than hormone-responsive tumors. Unfortunately, there are currently no efficacious biologically-based treatments for these more aggressive forms of breast cancer whose survival rates are less than 26%. Polyamines and ODC are increased in breast cancer compared with normal breast tissue, and increased ODC activity directly correlates with a less differentiated and more metastatic tumor phenotype [5,6,15]. ODC activity is also an independent adverse prognostic factor for overall breast cancer survival [5,6,15]. DFMO, an irreversible inhibitor of ODC, reduces polyamine pools resulting in a cytostatic effect. Our previous studies demonstrated that treatment with DFMO reduces lung metastasis of the hormone-independent MDA-MB-435 breast cancer cell line with only a modest effect on the growth of orthotopic tumors [8,16]. This led to two questions: Can inhibition of ODC activity by DFMO decrease metastasis of MDA-MB-435 cells to another secondary site, namely bone? Can DFMO decrease orthotopic tumor growth and lung metastasis of another hormone-independent breast cancer cell line, MDA-MB-231? DFMO inhibition of tumor growth and metastasis of hormone-independent cells would strongly support targeting the polyamine pathway as a potential treatment.

### Polyamine levels are decreased in tumors treated with DFMO

**Figure 1:**
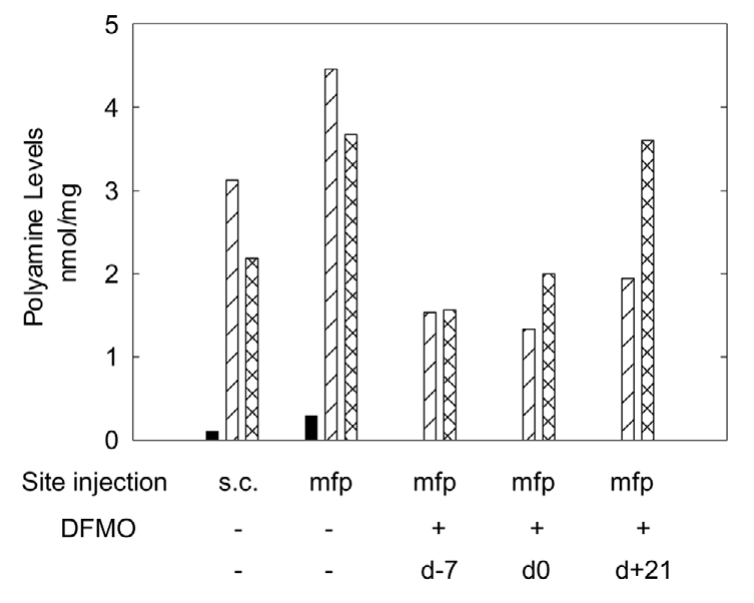
α-Difluoromethylornithine (DFMO) (2% per orally in drinking water) decreases polyamine levels in mammary fat pad tumors of MDA-MB-231 cells. Polyamine levels were determined in control subcutaneous (s.c.) and mammary fat pad (mfp) tumors as well as in DFMO-treated tumors grown in the mammary fat pad. DFMO treatment began at either 7 days prior to tumor cell injections (DFMO-7), on the day of injection (DFMO 0) or 21 days post injection (DFMO+21). Putrescine (black), spermidine (diagonal line) and spermine (cross-hatched) levels are graphed as nanomoles per milligram of tumor.

Polyamine levels were determined in orthotopically (mammary fat pad) and ectopically (subcutaneous) growing tumors in order to demonstrate that DFMO was inhibiting ODC activity. DFMO treatment beginning at either 7 days prior to injection (d-7), on the day of injection (d0) or 21 days following (d+21) injection of MDA-MB-231 cells into the mammary fat pad resulted in decreased putrescine and spermidine levels with only slight effects on spermine levels (Fig. 1). Putrescine levels decreased from an average of 0.200 nmol/mg tissue in the control samples to below the detection level in all of the DFMO-treated samples, while the spermidine levels decreased from a mean of 3.7905 nmol/mg in the untreated samples to 1.606 nmol/mg after treatment. As expected [17,18], the spermine levels decreased only slightly from an average of 2.931 nmol/mg in the untreated controls to 2.390 nmol/mg after treatment. This demonstrates that ODC was effectively inhibited by DFMO treatment.

Curiously, polyamine levels in MDA-MB-231 cells were significantly different when the cells were grown orthotopically versus those grown ectopically (Fig. 1). This result suggested that DFMO treatments might exert different effects on cells depending upon their location. The result is not entirely surprising since several previous experiments have shown that the biologic behavior of tumor cells can vary widely based upon the site of injection [12].

### DFMO treatment decreases bone metastasis by MDA-MB-435 cells

We previously showed that MDA-MB-435 metastasis was decreased by 74% to the lung when mice were treated with DFMO [9] despite no apparent change in local tumor invasion. That result indicated that DFMO was affecting the later stages of metastasis (i.e. colonization). Breast cancers commonly metastasize to bone [19]. While bone metastases are not directly responsible for most breast cancer deaths, they result in profoundly decreased quality of life. While current therapies exist to decrease osteolysis and reduce sequella of bone metastases, there are few options to diminish bone tumor burden [19,20].

Taking into account the importance of breast cancer metastasis to bone and observations that polyamine levels varied depending upon tumor cell location in the body, we therefore asked whether DFMO had an effect on bone metastasis. To do this, we employed a model recently developed by ourselves using GFP-labeled MDA-MB-435 cells [10,11]. This model allows for relatively rapid assessment of the tumor burden in bone and is not dependent solely upon radiographic imaging and histology, which are less sensitive and more laborious, respectively.

**Figure 2:**
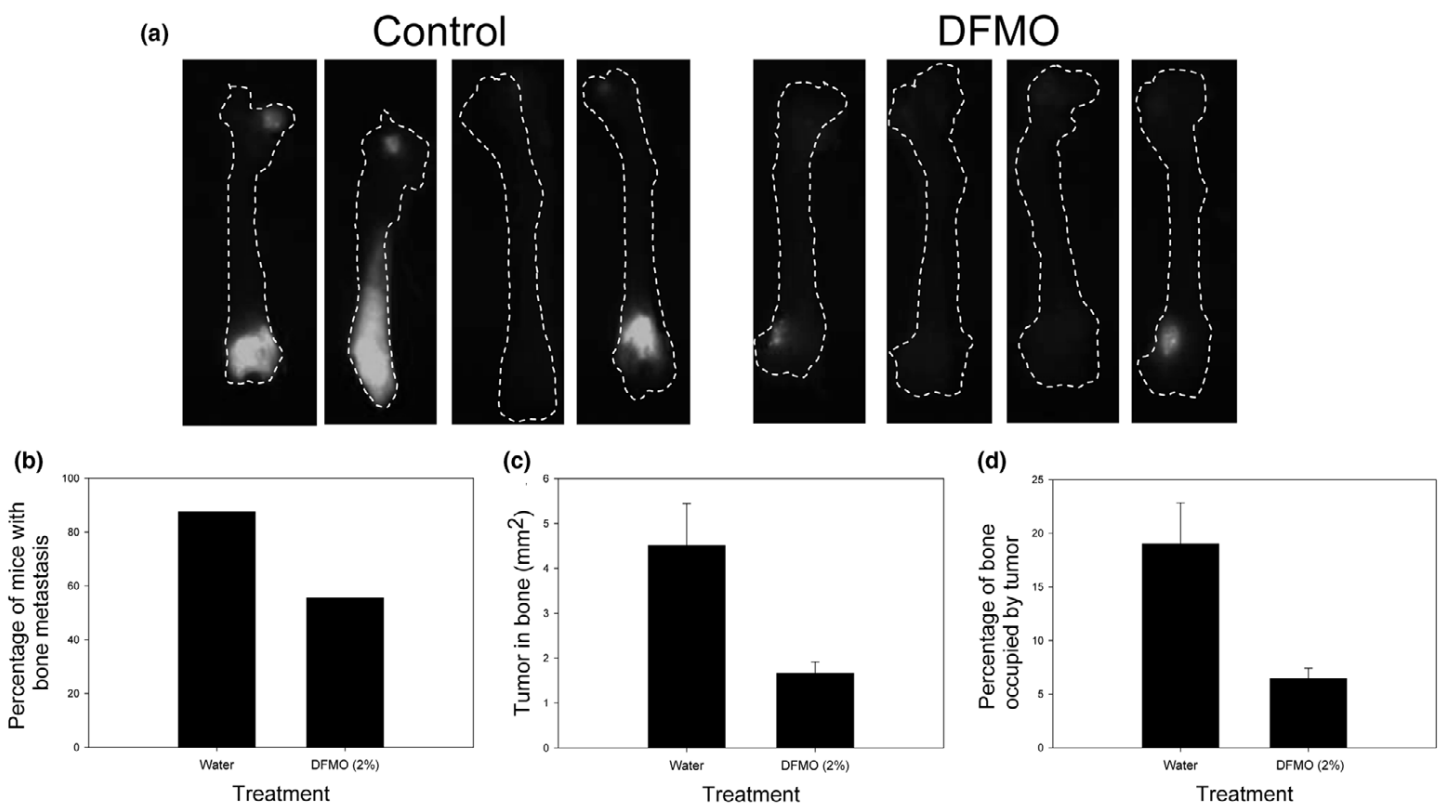
α-Difluoromethylornithine (DFMO) decreases the incidence and size of bone metastases from MDA-MB-435 cells. Green fluorescent protein-labeled MDA-MB-435 cells were injected intracardially into athymic mice. DFMO (2% per orally in drinking water *ad libitum*) treatment began 7 days prior to injection and continued for 6 weeks post injection. Bone metastases were visualized using a fluorescence microscope. **(a) **Photographs of femurs from control (*n *= 10) and DFMO-treated (*n *= 10) mice show the presence and size of the bone metastases. **(b) **Incidence of metastasis was determined by counting the number of mice with green fluorescence in the femur regardless of size of the mass. Image analysis was used to quantify fluorescence in each bone. **(c) **The area of tumor in bone was quantified by comparing the total area of the femur with the area containing green fluorescence. **(d) **The percentage of bone occupied by the tumor was also determined.

As for all xenograft models, metastases in bone are not observed following orthotopic injection; tumor cells are therefore injected directly into the left ventricle of the heart. While metastases develop in other bones, this study involved complete analysis only on femurs because this site reflects changes elsewhere [10,11] and because it is a site commonly affected in women with breast cancer. DFMO treatment was begun 7 days prior to intracardiac injection of GFP-labeled MDA-MB-435 cells and lasted for the duration of the experiment. Mice with fluorescently-labeled femoral tumors were counted (Fig. 2a,b). DFMO treatment decreased the number of mice with bone metastases from 87.5% to 55.5% (Fig. 2a,b). The GFP label in each femur was then quantified by image analysis and the area of bone occupied by tumor cells was calculated (Fig. 2c,d). DFMO significantly decreased the area of bone occupied by the tumor from 4.51 to 1.69 mm^2 ^(Fig. 2c; *P *< 0.05) and the percentage of bone occupied by the tumor decreased from 19.03% to 6.45% (Fig. 2d; *P *< 0.05). The intensity of fluorescence was not a variable for these analyses since we previously observed that intensity was dependent upon the depth of tumor cells to the bone surface [10]. As an internal control, however, bones were examined from both sides, and the areas occupied by tumor were found to be comparable (data not shown). Previous and ongoing independent studies have validated tumor location by histology and histomorphometry.

To the best of our knowledge, these are the first data to demonstrate efficacy of DFMO for metastases at a site other than the lung. The polyamine pathway therefore shows promise as a target for decreasing metastasis to multiple sites in breast cancer patients. Our studies have a modest limitation in that they only test efficacy of DFMO against the MDA-MB-435 cells to bone. We and other workers [21-23] have shown that the MDA-MB-231 cells will also colonize bone. But in the absence of GFP-tagged variants, the MDA-MB-231 bone metastasis assays are limited to radiologic or histologic detection methods that are less sensitive or more laborious. They were therefore not utilized here.

### MDA-MB-231 orthotopic tumor growth is delayed by DFMO

This study, combined with previous data, indicates that the polyamine pathway can be modulated to affect primary tumor growth and metastasis of a hormone-independent breast cancer cell line (MDA-MB-435). To begin assessing whether DFMO treatment might be generally efficacious against other hormone-independent breast cancers, we examined orthotopic tumor growth and lung colonization of a second metastatic hormone-independent breast cancer cell line, MDA-MB-231.

**Figure 3:**
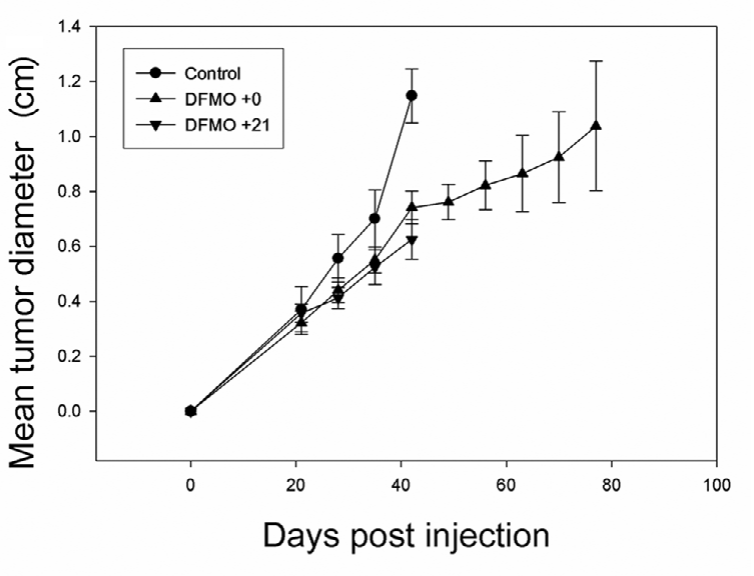
α-Difluoromethylornithine (DFMO) delays orthotopic tumor growth of MDA-MB-231 cells. Athymic mice were injected into the second thoracic mammary fat pad with MDA-MB-231 cells. DFMO treatment (2% per orally) began either contemporaneously (▴, *n *= 20) or 21 days post injection (▼, *n *= 3). Mammary fat pad tumor length and width were measured once weekly. Control (●, *n *= 17) and 21 days post injection (DFMO+21) animals were sacrificed 6 weeks post injection. Day of injection (DFMO 0) animals were euthanized either 6 weeks (*n *= 10) or 11 weeks (*n *= 10) post injection.

Orthotopic tumor growth of MDA-MB-435 cells was previously shown to be mildly, but significantly, delayed by or not affected by treatment with DFMO [8,9]. Untreated MDA-MB-435 tumors reached a size of 100 mm^2 ^2 weeks earlier than DFMO-treated tumors. We had also previously shown that MDA-MB-231 orthotopic tumors were delayed by DFMO treatment. We repeated this experiment with slight variations. DFMO treatment began either on the day of injection (d0) or 21 days post injection (d+21) of MDA-MB-231 cells into the second thoracic mammary fat pad and were continued until tumors reached a mean tumor diameter of approximately 1.2 cm (Experiment 1) or until 6 weeks post injection (Experiment 2). DFMO treatment delayed orthotopic tumor growth to ~1.2 cm from 42 days in control animals to 77 days (Fig. 3). At 42 days post injection, control tumors were 1.15 cm while DFMO-treated tumors were 0.75 cm when treatment began concurrent with tumor injection, and surprisingly smaller (0.62 cm) when treatment began on day 21 (Fig. 3).

DFMO treatment can thus delay orthotopic tumor growth of more than one breast cancer cell line, albeit with heterogeneous responses. MDA-MB-231 cells were more sensitive to DFMO at the primary site; that is, MDA-MB-435 tumors were mildly delayed (~2 weeks) or not delayed at all compared with a delay of ~5 weeks for MDA-MB-231. Postponement of DFMO treatment until 3 weeks post injection also inhibited MDA-MB-231 tumor growth, suggesting that DFMO could still be efficacious if administered at the time of diagnosis.

### MDA-MB-231 lung metastasis is decreased by DFMO

**Figure 4:**
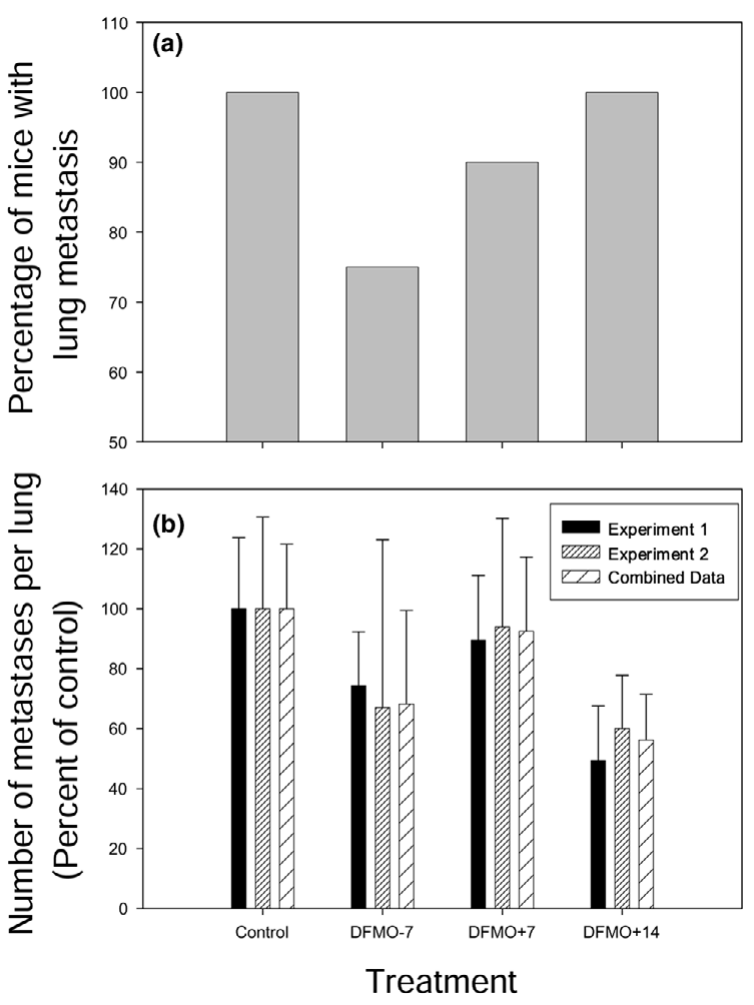
α-Difluoromethylornithine (DFMO) treatment decreases lung metastasis of MDA-MB-231 cells. MDA-MB-231 cells were injected into the tail vein of athymic mice. DFMO treatment (2% per orally) began either 7 days prior to (DFMO-7), or 7 days (DFMO+7) or 14 days (DMFO+14) after, tumor cell injection. Mice were killed either 4 weeks (Experiment 1) or 6 weeks post injection (Experiment 2). Lungs were removed and fixed in Bouin's fixative diluted 1:5 in neutral buffered formalin. Each lung was examined for the presence of surface metastases. **(a) **The incidence of mice with lung metastases was graphed as a percentage of the total number of mice (*n *= 10 for each group). **(b) **The average number of metastases per lung was graphed as a percentage of the control (*n *= 10 for each group). Experiment 1 (black) and Experiment 2 (small hatched) are graphed separately as well as combined (large hatched).

Like MDA-MB-435 cells, DFMO reduced lung metastasis of MDA-MB-231 cells. The latter, however, had to be evaluated following injection of cells directly into the lateral tail vein because the parental cells are not effective at spontaneous metastasis. This experimental design afforded examination of scheduling since all tumor cells were administered as a bolus. While lung metastasis was reduced in all treatment groups, we were surprised by the pattern of suppression, even though it was reproducible. The incidence of metastases (i.e. proportion of mice developing lung metastases) decreased as expected. Earlier treatment resulted in fewer mice with metastases: 25%, 10% and 0% decreased for treatments beginning d-7, d0 and d+14 (Fig. 4a). However, the mice receiving DFMO beginning on d+14 showed diminishment of the number of metastases per lung (42%, mean = 12.5/lung vs 22.2/lung for controls), compared with no effect for d+7 (mean = 20.5/lung) and 32% for d-7 (mean = 15.1/lung) treatment schedules (Fig. 4b). Despite the trend toward decreased lung metastasis, and in contrast to MDA-MB-435 cells, the reductions in lung metastasis never reached statistical significance (*P *< 0.05) for MDA-MB-231 cells. Nonetheless, DFMO was clearly exerting reproducible effects on breast carcinoma growth in the mammary fat pad and in the lung.

### DFMO treatment decreases proliferation in MDA-MB-231 orthotopic tumors

**Figure 5:**
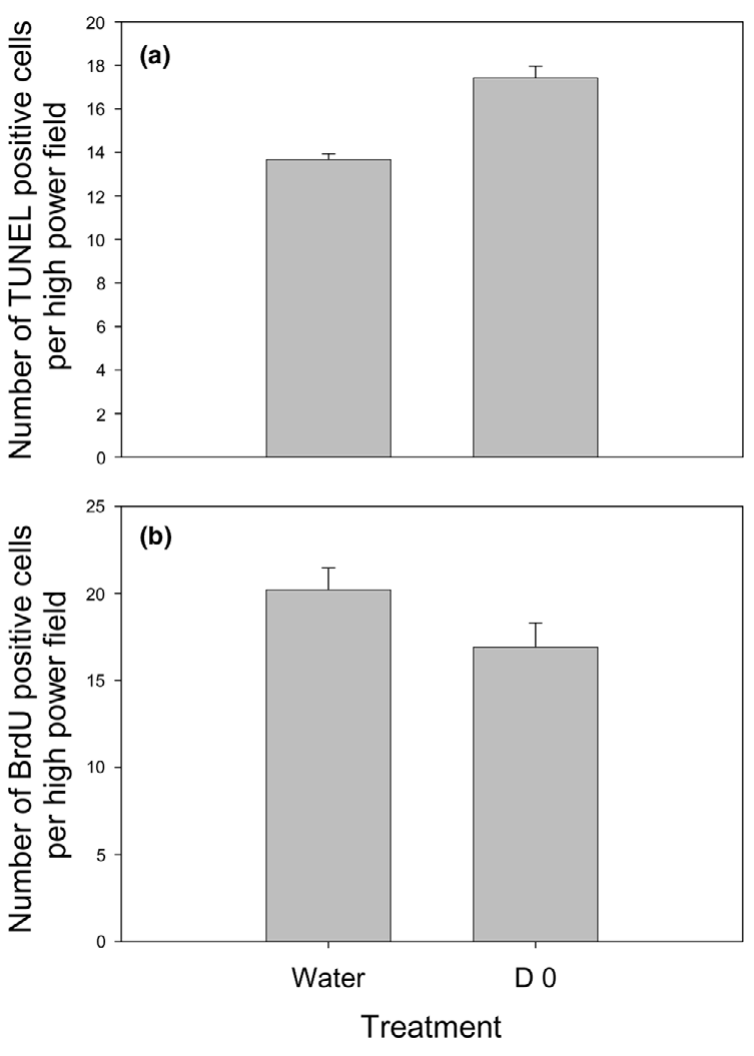
α-Difluoromethylornithine (DFMO) treatment affects both cell proliferation and cell death in MDA-MB-231 orthotopic tumors. **(a) **TUNEL analysis and **(b) **BrdU analysis of MDA-MB-231 orthotopic tumors from either control mice or mice treated with 2% DFMO in the drinking water beginning at the day of injection.

To begin addressing mechanisms for increased susceptibility of MDA-MB-435 cells to spontaneous metastasis suppression and of MDA-MB-231 cells to suppression of tumor growth, we performed additional studies. *In vitro *growth was not significantly affected (data not shown). To further assess growth and apoptosis, we examined MDA-MB-231 tumors growing orthotopically by BrdU and TUNEL staining to determine whether DFMO treatment altered proliferation or apoptosis, respectively. We found that DFMO treatment beginning d0 increased apoptosis (Fig. 5a) and slightly decreased proliferation (Fig. 5b) compared with control tumors. The decrease in BrdU labeling is consistent with, but less dramatic than, the 60% decrease of Ki67 labeling observed in DFMO-treated MDA-MB-435 tumors, while the increase in TUNEL staining is in contrast to the MDA-MB-435 tumors where no change in apoptosis was observed, even though cleaved caspase-3 levels were increased fourfold. Non-apoptotic necrosis was decreased by 60% in the MDA-MB-435 tumors, resulting in no change in overall tumor volume [24].

### Meprin α expression is affected by DFMO in MDA-MB-435 cells

Since we previously showed that DFMO does not affect local tumor invasion of either MDA-MB-231 or MDA-MB-435 cells, we reasoned that DFMO was exerting its major effect on later stages of metastasis. This conclusion incorrectly led us to hypothesize that proteinases were not (or only modestly) affected by DFMO treatment. However, recent evidence suggested that proteinases, and their corresponding inhibitors, have roles in tumor progression at steps other than invasion [25], prompting a closer look.

Meprin α is a zinc-dependent endopeptidase that is normally expressed in kidney and intestinal epithelial cells. It is found as either an apical membrane-bound form or as a secreted protein. At different stages of development, meprin α is expressed in a variety of tissues. *In vitro*, matrix proteins such as fibronectin, laminin and collagen are meprin α substrates. In some epithelial carcinomas, meprin α secretion can occur both apically and basolaterally, and is activated by plasmin released by stromal fibroblasts. Together these data indicate that meprin α can contribute to cancer invasion and/or metastasis.

**Figure 6:**
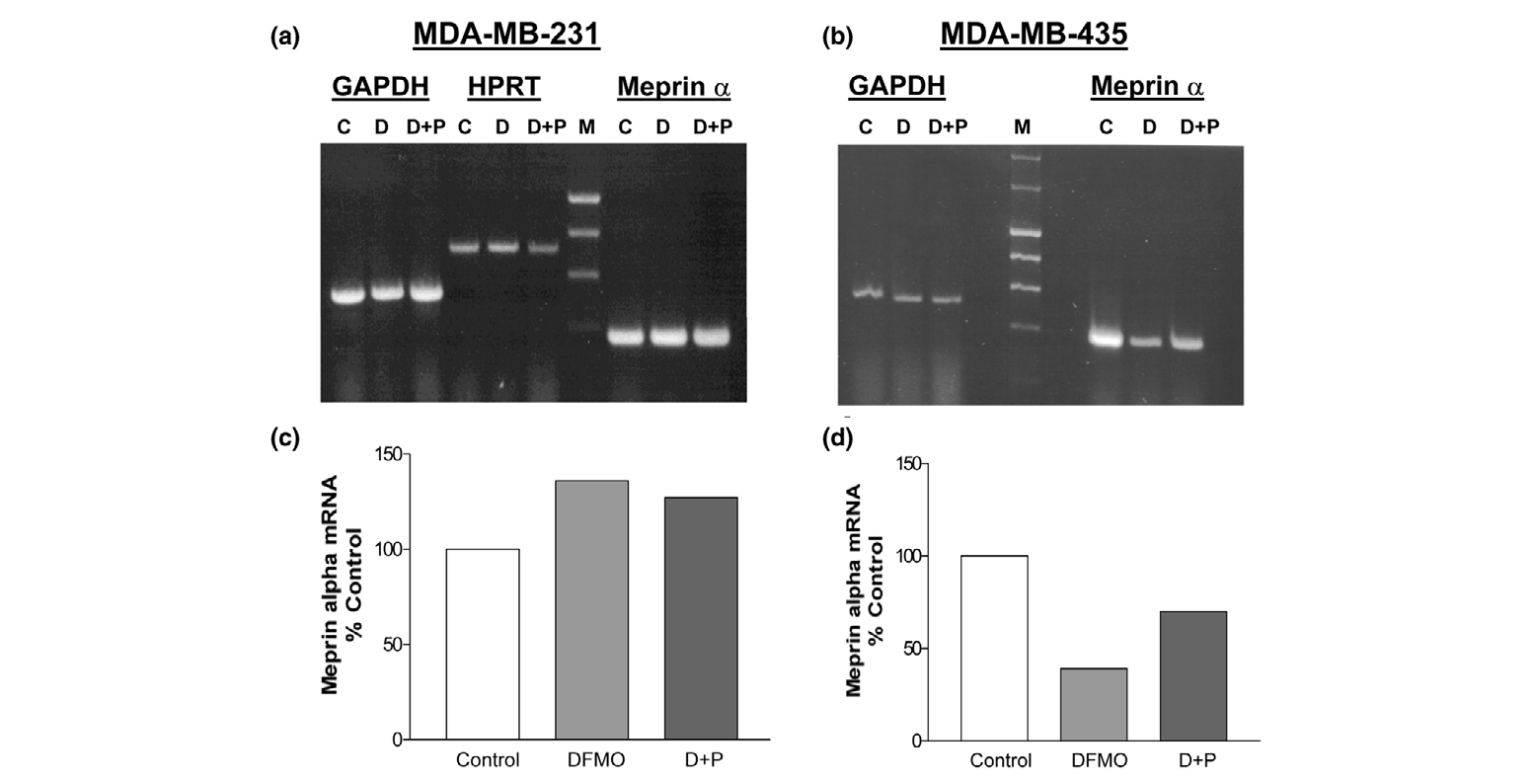
*In vitro *α-difluoromethylornithine (DFMO) treatment decreases meprin α expression in MDA-MB-435 cells, but not in MDA-MB-231 cells. End-point RT-PCR analysis of total RNA from **(a) **MDA-MB-231 cells or **(b) **MDA-MB-435 cells, either left untreated (C), treated with 1 mM DFMO for 48 hours (D) or treated with 1 mM DFMO plus 2.5 mM putrescine for 48 hours (D + P). Quantities of meprin α mRNA in **(c) **MDA-MB-231 cells or **(d) **MDA-MB-435 cells were normalized to glyceraldehyde-3-phosphate dehydrogenase levels (GADPH) – normalization to hypoxanthine phosphoribosyl transferase [HPRT] was also performed in (a). Meprin α expression levels are graphed as a percentage of control.

To assess the impact of DFMO treatment on meprin α, MDA-MB-435 and MDA-MB-231 cells were treated *in vitro *with 1 mM DFMO ± 2.5 mM putrescine (to demonstrate that the inhibition is specific for ODC) for 48 hours. Semi-quantitative RT-PCR showed that DFMO treatment had no effect on meprin α expression in MDA-MB-231 cells, while meprin α expression was decreased by greater than 50% in MDA-MB-435 cells (Fig. 6). Administration of putrescine partially reversed the effect of DFMO in MDA-MB-435 cells (Fig. 6b,d). Although not an exhaustive study, the meprin α results highlight the heterogeneity between different hormone-independent breast carcinomas with regard to their response to DFMO.

While there are likely to be other mechanisms involved, the difference in meprin α levels in DFMO-treated MDA-MB-231 and MDA-MB-435 cells provides at least a partial explanation for the different metastatic phenotypes of these cells in response to DFMO.

In support of our data, another inhibitor of polyamine pathway activity, SL11144, inhibited the growth of both MDA-MB-231 and MDA-MB-435 cells *in vitro *and of MDA-MB-231 orthotopic tumor growth *in vivo *[26]. *In vitro *cell death assays demonstrated a differential effect of SL11144 inhibition on the time line of apoptosis. Specifically, MDA-MB-435 cells exhibited significant DNA laddering at 12 hours after treatment, while MDA-MB-231 cells only showed the initial stages of laddering at 96 hours post-treatment. These results were explained by more potent activation of caspases and rapid cytochrome c release in MDA-MB-435 cells in response to the inhibitor compared with MDA-MB-231 cells. Similar to the studies reported here, SL11144 was effective against both cell lines, but the effect was different between the two lines.

Although heterogeneous responses were observed in the cell lines examined in the present study, the results highlight the potential utility of DFMO for the treatment of advanced hormone-independent breast cancers. Moreover, the finding that DFMO can significantly reduce bone metastasis warrants further studies into the utilization of polyamine pathway modulators in combination with other therapies for control of cancer metastasis.

## Conclusion

DFMO, a potent inhibitor of polyamine metabolism, blocks primary tumor growth and/or metastasis to the lung and bone from hormone-independent breast carcinoma xenografts. While the effects are heterogeneous, the findings warrant follow-up into the utilization of polyamine pathway modulators in combination with other therapies for control of this particularly difficult-to-treat class of breast carcinomas.

## Abbreviations

BSA = bovine serum albumin; DFMO = α-difluoromethylornithine; DMEM = Dubecco's modified Eagle's medium; GFP = green fluorescent protein; HBSS = Hanks Balanced Salt Solution; ODC = ornithine decarboxylase; PBS = phosphate-buffered saline; RT-PCR = reverse transcriptase-polymerase chain reaction; TUNEL = terminal deoxynucleotidyl transferase dUTP nick end labeling.

## Competing interests

The authors declare that they have no competing interests.

## Authors' contributions

MMR provided Figs 1, 3, 4 and 5, and drafted the manuscript. PAP and DJD provided Fig. 2. GM and JSB helped conceive of the meprin part of the study and provided Fig. 6. SW assisted with multiple technical aspects of this study. LMD performed the high-pressure liquid chromatography to determine polyamine levels. AM, MMR and DRW conceived of the study and helped to draft the manuscript. All authors have read and approved the final version of this manuscript.
